# X-Ray Single-Grating Interferometry for Wavefront Measurement and Correction of Hard X-Ray Nanofocusing Mirrors

**DOI:** 10.3390/s20247356

**Published:** 2020-12-21

**Authors:** Jumpei Yamada, Takato Inoue, Nami Nakamura, Takashi Kameshima, Kazuto Yamauchi, Satoshi Matsuyama, Makina Yabashi

**Affiliations:** 1 RIKEN SPring-8 Center, 1-1-1 Kouto, Sayo, Hyogo 679-5148, Japan; kameshima@spring8.or.jp (T.K.); yabashi@spring8.or.jp (M.Y.); 2 Division of Precision Engineering and Applied Physics, Graduate School of Engineering, Osaka University, 2-1 Yamada-oka, Suita, Osaka 565-0871, Japan; t.inoue@up.prec.eng.osaka-u.ac.jp (T.I.); nakamura@up.prec.eng.osaka-u.ac.jp (N.N.); yamauchi@prec.eng.osaka-u.ac.jp (K.Y.); matsuyama@prec.eng.osaka-u.ac.jp (S.M.); 3 Japan Synchrotron Radiation Research Institute, 1-1-1 Kouto, Sayo, Hyogo 679-5148, Japan; 4 Department of Materials Physics, Graduate School of Engineering, Nagoya University, Furo-cho, Chikusa, Nagoya 464-8603, Japan

**Keywords:** X-ray mirror, X-ray nanofocusing, wavefront correction, grating interferometer

## Abstract

X-ray single-grating interferometry was applied to conduct accurate wavefront corrections for hard X-ray nanofocusing mirrors. Systematic errors in the interferometer, originating from a grating, a detector, and alignment errors of the components, were carefully examined. Based on the measured wavefront errors, the mirror shapes were directly corrected using a differential deposition technique. The corrected X-ray focusing mirrors with a numerical aperture of 0.01 attained two-dimensionally diffraction-limited performance. The results of the correction indicate that the uncertainty of the wavefront measurement was less than *λ*/72 in root-mean-square value.

## 1. Introduction

The development of X-ray nanofocusing optics has rapidly progressed in the last two decades. Sub-10 nm focusing spots have been achieved using various types of X-ray optics [1,2,3,4], and the challenges of realizing ~1 nm spot sizes are presently being undertaken [5]. Through the competition of the tiny X-ray focusing, the numerical aperture (NA) of the focusing optic has increased, and the optical fabrication with sufficient fidelity has become challenging. In the case of a reflective X-ray focusing mirror, a high-NA hard X-ray mirror for the tiny focusing has a steeply curved surface with a radius of curvature (RoC) of a few meters [6,7]. It has been difficult to precisely measure such a small-RoC surface by conventional metrology, but the required shape accuracy for the mirror is less than 1 nm in peak-to-valley (PV) value.

Recently, X-ray wavefront correction (XWC) schemes have been investigated intently with several approaches [6,7,8,9,10]. In those schemes, wavefronts of the focused X-ray beams are characterized by wavefront sensors (WFSs), and subsequently, additional optics or treatments for optics are adopted to compensate for the wavefront distortions. The X-ray WFS is applicable to mirrors even with a small RoC and can provide an accuracy better than 1 nm PV due to the short-wavelength metrology. XWC has thus been a promising way to overcome the difficulty in fabricating the high-NA and small-RoC hard X-ray mirrors [6,7].

The reachable accuracy of the XWC is limited by the WFS. Among many types of WFSs [10,11,12,13,14,15,16,17,18,19], a single-grating interferometer (s-GI) using the Talbot effect is regarded as one of the fast and less-chromatic tools [20,21,22,23,24,25]. The s-GI comprises a simple arrangement using only two optics of a grating and an X-ray detector. Although the small number of components is favorable for achieving high accuracy in the wavefront sensing theoretically, s-GIs have still suffered from systematic errors in determining the accurate wavefront distortion [7,26,27]. Calibration methods using an adequately small pinhole have been employed in extreme ultraviolet (EUV) lateral shearing interferometers and X-ray WFSs for larger foci than 100~200 nm [24,25]. In the aforementioned hard X-ray nanofocusing, however, the required diameter of the pinhole is smaller than 1~10nm, which is difficult to fabricate with a sufficient thickness. A few methods to calibrate the systematic error of the detector have been reported [14,25,26], but comprehensive study is necessary to ensure an accuracy of XWC that enables fine nanofocusing and conducting reliable X-ray experiments.

In this letter, we describe the results of a study of accurate X-ray wavefront measurements using the s-GI, which were exploited for hard X-ray focusing mirrors with an NA of 0.01 [28]. We carefully examined the systematic errors caused by a grating fidelity, a detector distortion, and alignment errors of the components. Finally, we describe the results of XWC for the mirrors, which was implemented using a shape correction technique of a differential deposition [29]. The correction results indicate that the uncertainty of our s-GI was less than *λ*/72 root-mean-square (rms).

## 2. Materials and Methods

**Figure 1:**
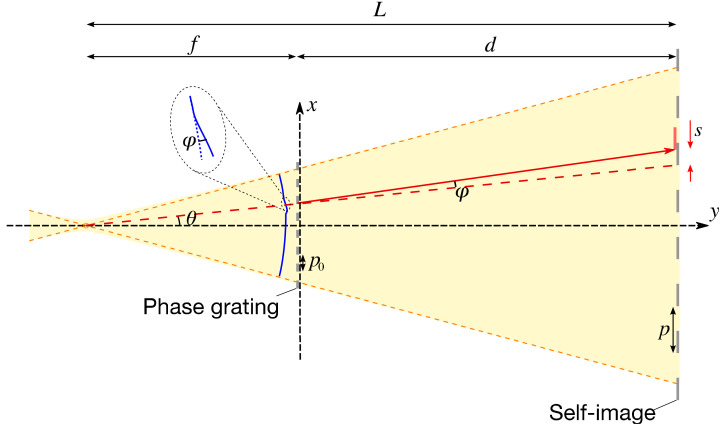
Schematic illustration of the single-grating interferometer and ray trajectory with a local wavefront distortion.

When an amplitude or phase grating is illuminated by a fully or partially coherent X-ray beam diverging from a focal point, as illustrated in Figure 1, magnified periodic intensity patterns corresponding to the grating period—so-called self-images—are generated at specific downstream positions. The relationship between a distance from the focus to the self-image *L* and a distance from the focus to the grating *f* can be written, as Yashiro et al. reported [30], as

(1) $$L=\;\frac{f^{2}}{f-m\frac{p_{0}{}^{2}}{\lambda }}\;$$

or

(2) $$f=\frac{L}{2}\left(1\pm \sqrt{1-\frac{4mp_{0}{}^{2}}{L\lambda }}\right)\;,$$

where the *p*_0_ is the period of the grating, *λ* is the wavelength, and *m* is the Talbot order. Equation (2) possesses two solutions, which indicates that the grating should be placed near the focus or far from focus (near the detector). The former case has been generally employed for the wavefront sensing of the focused X-ray beam because of high angular sensitivity.

For the wavefront sensing, one can measure the self-image and analyze it using the following procedure, with experimental parameters of *p*_0_, *λ*, a distance from the grating to the detector *d*, and a pixel size of the detector *∆x*’. First, one can measure a period of the self-image *p* from the acquired image. The RoC of the wavefront at the grating plane *R* (i.e., effective *f* including focal shifts and arrangement errors) is given by

(3) $$R=\;\frac{dp_{0}}{p-p_{0}}\;,$$

which corresponds to the quadratic wavefront term.

Next, one can dissect the self-image by the Fourier transform method (FTM) [31] or the fringe-scanning method (FSM) [32]. Both methods fundamentally give the same information of a fringe-phase *ψ*(*x*), which indicates a distortion of the self-image with a radian unit. The FTM generally extracts the adjacent spectrum to the zero-order peak in the reciprocal space. Note that this inherently works as bandpass filtering, corresponding to a reduction of errors due to high-order diffraction harmonics that are involved in a nonsinusoidal fringe pattern. The FSM may also include the error from high-order harmonics. It is known that adequately large-number-step scanning or five-step scanning with the Ronchi (line–space ratio of 1:1) gratings can reduce the error [33,34].

Using the fringe-phase *ψ*(*x*), deviation of the fringes *s*(*x*), which stems from the distorted wavefront, can be written as

(4) $$s\left(x\right)=\;\frac{p_{0}}{2\pi }\frac{L}{f}\psi \left(x\right)\;.$$

Using the deviation angle of distorted wavefront slope *φ*(*x*), which is equivalent to the deviation angle of the ray, the *s*(*x*) can be also expressed as

(5) $$s\left(x\right)=d\{\tan\left[\theta \left(x\right)+\varphi \left(x\right)\right]-\tan\theta \left(x\right)\}\;,$$

where the *θ*(*x*) denotes an ideal divergent angle from the focal point. Using the addition formula of the tangent, Equation (5) can be rewritten as,

(6) $$\tan\varphi \left(x\right)=\frac{s\left(x\right)}{d+s\left(x\right)\tan\theta \left(x\right)+d\tan^{2}\theta \left(x\right)}\;.$$

The differential of wavefront *∂*Φ(*x*)*/∂x* is given by

(7) $$\frac{\partial \Phi \left(x\right)}{\partial x}=\frac{2\pi }{\lambda }\tan\varphi \left(x\right)\;,$$

and then, from Equations (4), (6), and (7), the wavefront Φ(*x*) is given by

(8) $$\begin{array}{c} \Phi \left(x\right)=\frac{p_{0}}{\lambda }\;\frac{L}{f}\int \psi \left(x\right)\frac{1}{d+a\left(x\right)}\;dx\\ a\left(x\right)=s\left(x\right)\tan\theta \left(x\right)+\;d\tan^{2}\theta \left(x\right)\;. \end{array}$$

The sin*θ*(*x*) takes the NA value of the X-ray focusing system in maximum. If the NA is sufficiently small, the sin^2^*θ*(*x*) ≈ tan^2^*θ*(*x*) is close to 0. The *s*(*x*) is also generally small (1 × 10^−5^~1 × 10^−4^), so the *a*(*x*) term can be ignored. Using an effective pixel size at the grating plane *∆x* = *∆x’f*/*L*, the Φ*(x)* can be derived consequently as

(9) $$\Phi \left(x\right)\approx \frac{p_{0}}{d\lambda }\;\frac{L}{f}\int \psi \left(x\right)\;dx\approx \frac{p_{0}\Delta x^{\prime }}{d\lambda }\underset{i=0}{\sum }\psi \left(x_{i}\right)\;,$$

where *i* denotes an integer unit of the data. Equation (9) results in the same expression as in the case of parallel beam illumination (Fresnel scaling theorem). In this study, the s-GI is described by a ray-tracing manner with the self-image precondition for clarity, but a wave-optical description returns almost the same expression [20,30,35]. The required spatial coherence length for the Talbot effect is approximately *mp*_0_ [34], resulting in 2~10 µm generally, because interference between neighboring diffraction orders of grating forms the sinusoidal profile of the self-image. It is well known that temporal coherency, in other words, monochromaticity, is not strictly required (e.g., *∆λ/λ* < 10~20%) for both the fringe visibility and the measurement accuracy [34,36]. For the complete wavefront characterization, a two-dimensional (2D) integration procedure is necessary. Huang and Idir et al. [37] thoroughly investigated the 2D integration and suggested that one can choose a suitable integration method depending on requirements of a calculation speed, accuracy, shape of a pupil, etc.

In this work, the wavefront sensing using the s-GI for the tightly focused X-ray beams was performed at the 1 km experimental station of SPring-8 BL29XU [38] at a photon energy of 9.1 keV. We utilized one-dimensional (1D) *π*/2-phase gratings with a period of 2.5 µm and a 2D checker-board *π*-phase grating with a period of 3.0 µm (NTT Advanced Technology Co.). Both 1D and 2D gratings were made of Ta and fabricated by electron beam lithography, which is a well-known nanofabrication method. The 2D *π*-phase grating generated a self-image corresponding to half of the grating period, which could facilitate the high-spatial-resolution measurements. We adopted the FTM for the fringe analyses and the cosine transform integration [37,39] for the 2D integration, in which fast calculations were available. For the detector, scintillator-lens-coupled X-ray sCMOS cameras were utilized. Details of the detector configurations were described in Section 3.2. For the focusing, advanced Kirkpatrick–Baez mirror optics based on the Wolter type III geometry [40,41] were exploited, which were developed for the nanofocusing of X-ray free-electron laser (XFEL) pulses [28]. The mirrors had an NA of 0.01 and were designed for focusing at 9.1 keV. The camera distance *L* was set to be 0.495 m, and thus the *f* was 11.7 mm with Talbot order of 1/4 for the 1D grating and 8.4 mm with the Talbot order of 1/8 for the 2D grating. Note that the influence of the *a*(*x*) term in Equation (8) was less than *λ*/300 PV in the present case. Through the results presented in this study, the quadratic wavefront terms were subtracted to calculate wavefront errors because they were not critical for the XWC of the mirrors and can be evaluated by the RoC of the wavefronts.

## 3. Results and Discussion

From Equation (9), it can be seen that undesired distributions of the *p*_0_, *∆x*’, *d*, *λ* generate the systematic errors in the s-GI. Since the wavelength *λ* was monochromatized by a double-crystal Si(111) monochromator (*∆λ/λ* < 2 × 10^−4^) which was well calibrated with a photon energy accuracy of ~2eV by using the Zr absorption edge, we examined the remaining three factors. Then, the XWC was performed, and the certainty of the s-GI was estimated.

### 3.1. Fidelity of the Grating

**Figure 2:**
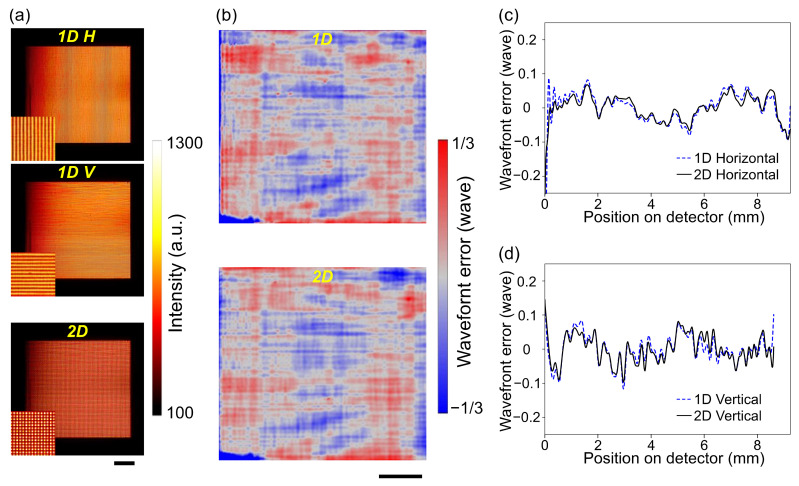
Comparison of the 1D and 2D gratings. (**a**) Obtained self-images with the 1D horizontal (top), 1D vertical (middle), and 2D checker-board (bottom) gratings. Insets show the zoom-in views of 2 × 2 mm^2^ center areas. (**b**) Wavefront errors at the detector plane measured using the 1D (top) and the 2D (bottom) gratings. (**c**,**d**) Comparisons of the averaged profiles in the horizontal (**c**) and vertical (**d**) directions. All bars denote 2 mm.

The systematic error caused by the nonuniformity of grating period *p*_0_ was investigated. First, the difference between the 1D and 2D gratings was characterized. The acquired self-images of 1D and 2D gratings are shown in Figure 2a. The periods of the self-images were sufficiently fine, which led to the spatial resolution of the s-GI with the FTM analysis. The wavefront errors measured by the 1D and 2D gratings are shown in Figure 2b–d. It should be noted here that, since two gratings were utilized for measurement by 1D gratings (Figure 2b top), slight differences and local defects of the gratings gave mismatched information in horizontal and vertical phase gradients, resulting in integration errors. The difference between wavefronts measured by 1D and 2D gratings was less than *λ*/18 rms in 2D data, *λ*/30 rms in the horizontal profiles, and *λ*/50 rms in the vertical profiles. The results indicated that 2D grating was fabricated with sufficient accuracy, at least almost same as the 1D gratings, despite the relatively thick and complicated structures. For the fast characterization, the 2D grating was employed for the remaining studies.

**Figure 3:**
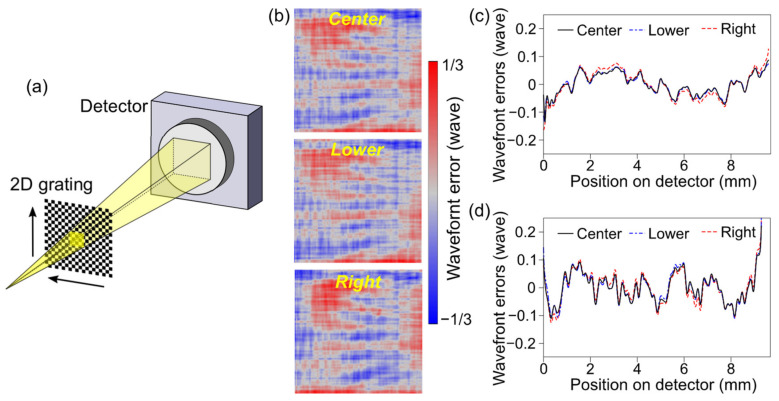
Dependences of illumination area on the 2D grating. (**a**) Schematic drawing of the measurement setup. The 2D grating was laterally shifted with a 300 µm step in the horizontal and vertical direction. (**b**) 2D results of the wavefront errors measured using center (top), lower (middle), and right (bottom) area of the grating. (**c**,**d**) Averaged profiles in the horizontal (**c**) and vertical (**d**) directions.

Next, the uniformity of the 2D grating was examined. The wavefront errors were measured using laterally 300 µm shifted positions of the grating, as illustrated in Figure 3a, while the grating size was 1 mm square and the illumination area on the grating was approximately 170 µm square. The obtained wavefront errors of the center, vertically shifted (lower), and horizontally shifted (right) positions were almost identical (see Figure 3b–d). The differences from the averaged data were less than *λ*/37 rms in the 2D data, *λ*/130 rms in the horizontal profiles, and *λ*/110 rms in the vertical profiles, which were much smaller than the Maréchal’s criterion [42]. We concluded that the 2D grating was fabricated with sufficient uniformity, and the systematic error derived from the grating was negligible.

### 3.2. Distortion of the X-Ray Detector

**Figure 4:**
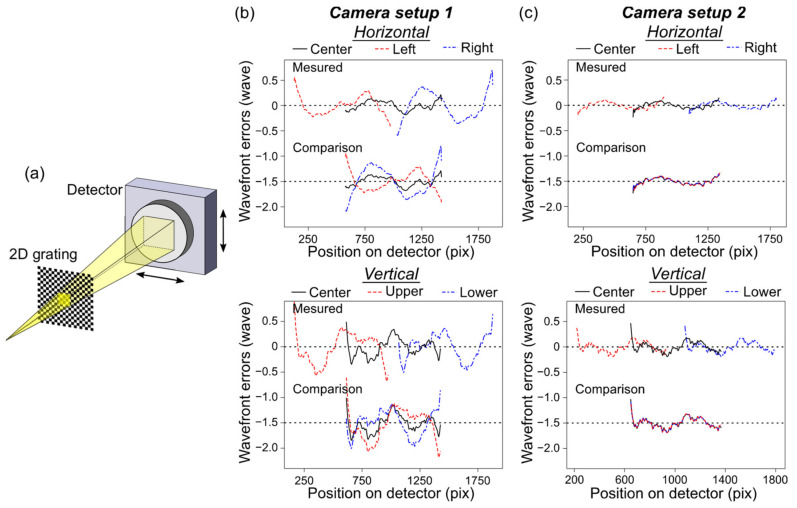
Dependences of the detector position. (**a**) Schematic drawing of the measurement setup. The detectors were laterally shifted in the horizontal and vertical directions. Two camera setups described in the main text were utilized. (**b**,**c**) Averaged wavefront error profiles along the horizontal (top) and vertical (bottom) directions, which were obtained using camera setup 1 (**b**) and 2 (**c**). For clarity, comparison profiles are shifted by −1.5 waves in (**b**) and (**c**).

The systematic error caused by the distortion of the X-ray detector, i.e., the distribution of the pixel size *∆x*’, was investigated. As illustrated in Figure 4a, the detector position dependences were examined by shifting the detectors in a similar way to the aforementioned grating uniformity test. Figure 4b shows the strong contribution of the distortion in the wavefront errors, which were measured using our conventional detector system (effective pixel size of 13.3 µm, 2048 × 2048 pixels) (camera setup 1), the same as the detector utilized in Ref. [7]. As some previous studies [14,26] reported that an aberration of distortion in the detector system significantly affected the wavefront measurements, we developed another detector system (camera setup 2). The camera setup 2 consisted of a low-distortion lens (MYUTRON Inc., LS05C, 0.5× magnification), a 0.7-mm-thick LuAG:Ce scintillator (Konoshima Chemical Co., Ltd.), and a sCMOS sensor (ORCA-Flash4.0 v2, Hamamatsu Photonics K.K., sensor size of 6.5 µm, 2048 × 2048 pixels). The detector position dependences, which were obtained again using the camera setup 2, are shown in Figure 4c. Owing to the corrected detector distortion, the wavefront error profiles measured using each position were quite consistent, and the discrepancy was less than *λ*/18 PV (*λ*/140 rms) in the horizontal and *λ*/20 PV (*λ*/160 rms) in the vertical direction. It was revealed that the appropriate visible-light lens could provide the nearly systematic-error-free X-ray detector system for the s-GI.

### 3.3. Alignments of the Grating and the Detector

**Figure 5:**
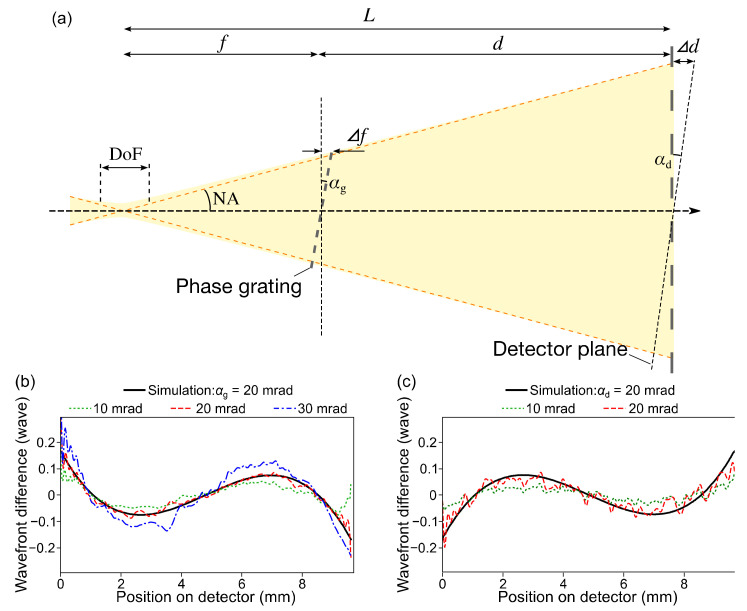
(**a**) Schematic illustration of the single-grating interferometer (s-GI) when the tilt errors of the grating and the detector are induced. (**b**,**c**) Results of the experiments and simulations for investigating the influence of tilt errors of the grating (**b**) and the detector (**c**).

Alignment errors, especially transverse tilt errors, of the grating and the detector inherently generate the unwanted variation of the distance from the grating to the detector *d* and cause the systematic errors. The influences of tilt errors of grating *α*_g_ and of detector *α*_d_, defined in Figure 5a, were investigated by purposely inducing the tilts. Moreover, numerical simulations based on the Fresnel–Kirchhoff’s diffraction integral [42], which mimicked the experimental conditions, were performed. Figure 5b and Figure 5c represent the experimental and simulation results for the *α*_g_ and *α*_d_, respectively. Here, the differences in the measured wavefront errors from those of the initial tilt conditions were plotted. The *α*_g_ and *α*_d_ of 20~30 mrad produced significant systematic errors, which were also verified by the simulation results. It is noteworthy that the third-order wavefronts, corresponding to the comatic aberration term, were deteriorated, which leads to confusion in alignment tuning of the X-ray focusing mirrors.

The tolerances for the tilt errors can be considered as follows. When the *α*_g_ is induced, the maximum displacement of the grating position ⊿*f* can be expressed using the small-angle approximation of the NA, as

(10) $$\Delta f\approx f\mathrm{NA}\;\tan\alpha_{g}\;.$$

If the ⊿*f* is much smaller than the depth of focus (DoF) of *λ*/NA^2^, it can be qualitatively considered irrelevant. Thus, the relationship of

(11) $$\begin{array}{l} f\mathrm{NA}\;\tan\alpha_{g}<k\frac{\lambda }{{\;\mathrm{NA}}^{2}}\;\\ \Rightarrow \;\tan\alpha_{g}<k\;\frac{\lambda }{f{\;\mathrm{NA}}^{3}}\; \end{array}$$

can be written, where *k* denotes the factor for acceptable systematic error amount. The expression regarding *α*_d_ can be derived, using the ⊿*d ≈ L*NAtan*α*_d_ and a revised demagnification factor of *f*/(*L* + ⊿*d)*, as

(12) $$\begin{array}{c} f\left(\frac{\mathrm{NA}\tan\alpha_{d}}{1+\mathrm{NA}\tan\alpha_{d}}\right)<k\frac{\lambda }{{\mathrm{NA}}^{2}}\\ \Rightarrow \;\tan\alpha_{d}<k\;\frac{\lambda }{f\;{\mathrm{NA}}^{3}}\;\;\left(\mathrm{NA}\ll 1\right)\;. \end{array}$$

The tolerances are inversely proportional to the cubic of NA. Hence, the high-NA X-ray focusing requires increasingly severe alignment accuracy so as to suppress the systematic error sufficiently. As a side note, if one follows Rayleigh’s quarter-wavelength rule [42], one should set the factor *k* to be approximately 2.0, as estimated by the simulation and experimental results.

In the present case, we pursued the reliable measurements and thus set the tolerances of the tilt errors to be 5 mrad, corresponding to the *k* of ~0.3. Using a reference cube prism, a tilt sensor, and accuracy-enhanced sample and detector mountings, we precisely aligned the grating and the detector within 3~4 mrad accuracy.

### 3.4. Wavefront Correction

**Figure 6:**
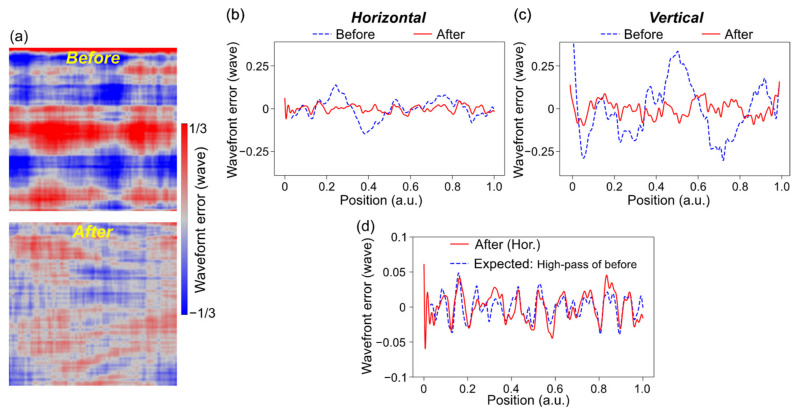
Results of the X-ray wavefront correction (XWC). (**a**) 2D wavefront errors originating from shape errors on the focusing mirrors before (top) and after (bottom) the correction. (**b**,**c**) 1D wavefront error profiles in the horizontal (**b**) and vertical (**c**) directions. (**d**) Comparison between the horizontal profiles of the corrected wavefront and of the expected wavefront, which is the high-pass filtered profiles before the correction.

After reducing the systematic errors and tuning the focusing mirror alignments, the wavefront error originating from shape errors on the mirrors could be deduced. The shape errors were derived using the designed distribution of incident angles to mirrors and a relationship of Φ = 2 *d*_s_sin*θ*_inc_, where the *d*_s_ denotes the shape error of mirrors and *θ*_inc_ denotes the glancing incident angle [42]. For the XWC, the shape errors were corrected using the differential deposition [7,29], which was an ex-situ precise additive shaping technique, by arbitrarily controlling the coating layer thickness in the magnetron sputtering deposition. The correction amounts for the horizontal and vertical mirrors were calculated by low-pass-filtering the measured shape errors, due to the limited spatial resolution of the differential deposition. The results of the XWC are shown in Figure 6. The 2D wavefront errors were improved to less than *λ*/15 rms, which indicated the capability for diffraction-limited focusing. In particular, the comparison between the corrected and expected profiles along the horizontal direction (Figure 6d) demonstrated that the XWC was almost perfectly accomplished. The difference of the profiles, which included the inaccuracy of the shape correction, was less than *λ*/72 rms, guaranteeing the certainty of the s-GI measurements.

## 4. Conclusions

In this study, we established a highly accurate s-GI by carefully investigating the origin of the systematic errors. To the best of our knowledge, the XWC demonstrated in this work showed the most precise fabrication of the high-NA and small-RoC hard X-ray focusing mirrors in which the required accuracy could not be managed by conventional optical metrology. The s-GI using FTM analysis facilitated the fast measurement with a single data acquisition, which led to a capability for single-shot wavefront sensing at XFEL sources. We may need to consider damages to the grating in the case of XFELs, but in our past experiments [7], the grating made of Ta placed in the defocused position barely survived the non-attenuated irradiations without damage and deformation. Attenuation of the XFEL pulses is certainly a reasonable way, and a grating made of Si or diamond would provide appropriate optics. The developed distortion-corrected X-ray detector system will be profitable for other WFSs such as Talbot (double-grating) interferometers [11], speckle-tracking methods [13,14], and Hartmann sensors [12]. The precise alignments of the components will be needed if the s-GI is applied for the focus characterization and/or the tuning of mirrors. In particular, the tolerance becomes remarkably stringent in the case of the NA higher than 0.02~0.03, which is required for a ~1 nm spot size or nanofocusing at soft X-ray wavelengths. Assessing and calibrating the quantitative comatic aberration by other methods will be an effective solution. A Ronchi shearing method [15], a ptychographic probe measurement [16,17], and a propagation-based wavefront reconstruction [18,19] will be applicable, in which also the quadratic wavefront term can be calibrated.

Continuous developments of the X-ray nanofocusing optics are a driving force for advancements of high-resolution X-ray analyses and explorations of novel X-ray sciences in synchrotron radiation and XFEL sources. We believe that the s-GI and the XWC demonstrated in this work will be paradigms for ultraprecise measurements and fabrications of the high-NA hard X-ray mirrors and stimulate further progress of X-ray nanofocusing optics.

## References

[1] Yamauchi K., Mimura H., Kimura T., Yumoto H., Handa S., Matsuyama S., Arima K., Sano Y., Yamamura K., Inagaki K. (2011). Single-nanometer focusing of hard x-rays by Kirkpatrick–Baez mirrors. J. Phys. Condens. Matter.

[2] Döring F., Robisch A.L., Eberl C., Osterhoff M., Ruhlandt A., Liese T., Schlenkrich F., Hoffmann S., Bartels M., Salditt T. (2013). Sub-5 nm hard x-ray point focusing by a combined Kirkpatrick-Baez mirror and multilayer zone plate. Opt. Express.

[3] Mohacsi I., Vartiainen I., Rösner B., Guizar-Sicairos M., Guzenko V.A., McNulty I., Winarski R., Holt M.V., David C. (2017). Interlaced zone plate optics for hard X-ray imaging in the 10 nm range. Sci. Rep..

[4] Bajt S., Prasciolu M., Fleckenstein H., Domaracký M., Chapman H.N., Morgan A.J., Yefanov O., Messerschmidt M., Du Y., Murray K.T. (2018). X-ray focusing with efficient high-NA multilayer Laue lenses. Light Sci. Appl..

[5] Chapman H.N., Bajt S. (2020). A ray-trace analysis of x-ray multilayer Laue lenses for nanometer focusing. J. Opt..

[6] Mimura H., Handa S., Kimura T., Yumoto H., Yamakawa D., Yokoyama H., Matsuyama S., Inagaki K., Yamamura K., Sano Y. (2010). Breaking the 10 nm barrier in hard-X-ray focusing. Nat. Phys..

[7] Matsuyama S., Inoue T., Yamada J., Kim J., Yumoto H., Inubushi Y., Osaka T., Inoue I., Koyama T., Tono K. (2018). Nanofocusing of X-ray free-electron laser using wavefront-corrected multilayer focusing mirrors. Sci. Rep..

[8] Kimura T., Handa S., Mimura H., Yumoto H., Yamakawa D., Matsuyama S., Inagaki K., Sano Y., Tamasaku K., Nishino Y. (2009). Wavefront control system for phase compensation in hard X-ray optics. Jpn. J. Appl. Phys..

[9] Seiboth F., Schropp A., Scholz M., Wittwer F., Rödel C., Wünsche M., Ullsperger T., Nolte S., Rahomäki J., Parfeniukas K. (2017). Perfect X-ray focusing via fitting corrective glasses to aberrated optics. Nat. Commun..

[10] Laundy D., Dhamgaye V., Moxham T., Sawhney K. (2019). Adaptable refractive correctors for x-ray optics. Optica.

[11] Weitkamp T., Nöhammer B., Diaz A., David C. (2005). X-ray wavefront analysis and optics characterization with a grating interferometer. Appl. Phys. Lett..

[12] Idir M., Mercere P., Modi M.H., Dovillaire G., Levecq X., Bucourt S., Escolano L., Sauvageot P. (2006). X-ray active mirror coupled with a Hartmann wavefront sensor. Nucl. Instrum. Methods Phys. Res. A.

[13] Bérujon S., Ziegler E., Cerbino R., Peverini R. (2012). Two-Dimensional X-Ray beam phase sensing. Phys. Rev. Lett..

[14] Berujon S., Ziegler E., Clotens P. (2015). X-ray pulse wavefront metrology using speckle tracking. J. Synchrotron Rad..

[15] Uhlén F., Rahomäki J., Nilsson D., Seiboth F., Sanz C., Wagner U., Rau C., Schroer C.G., Vogt U. (2014). Ronchi test for characterization of X-ray nanofocusing optics and beamlines. J. Synchrotron Rad..

[16] Maiden A.M., Rodenburg J.M. (2009). An improved ptychographical phase retrieval algorithm for diffractive imaging. Ultramicroscopy.

[17] Kewish C.M., Thibault P., Dierolf M., Bunk O., Menzel A., Vila-Comamala J., Jefimovs K., Pfeiffer F. (2010). Ptychographic characterization of the wavefield in the focus of reflective hard X-ray optics. Ultramicroscopy.

[18] Hagemann J., Robisch A.-L., Luke D.R., Homann C., Hohage T., Clotens P., Suhonen H., Salditt T. (2014). Reconstruction of wave front and object for inline holography from a set of detection planes. Opt. Express.

[19] Hagemann J., Robisch A.-L., Osterhoff M., Salditt T. (2017). Probe reconstruction for holographic X-ray imaging. J. Synchrotron Rad..

[20] Ray-Chaudhuri A.K., Ng W., Cerrina F., Tan Z., Bjorkholm J., Tennant D., Spector S.J. (1995). Alignment of a multilayer-coated imaging system using extreme ultraviolet Foucault and Ronchi interferometric testing. J. Vac. Sci. Technol. B.

[21] Naulleau P.P., Goldberg K.A., Bokor J. (2000). Extreme ultraviolet carrier-frequency shearing interferometry of a lithographic four-mirror optical system. J. Vac. Sci. Technol. B.

[22] Matsuyama S., Yokoyama H., Fukui R., Kohmura Y., Tamasaku K., Yabashi M., Yashiro W., Momose A., Ishikawa T., Yamauchi K. (2012). Wavefront measurement for a hard-X-ray nanobeam using single-grating interferometry. Opt. Express.

[23] Merthe D.J., Yashchuk V.V., Goldberg K.A., Kunz M., Tamura N., McKinney W.R., Artemiev N.A., Celestre R.S., Morrison G.Y., Anderson E.H. (2013). Methodology for optimal in situ alignment and setting of bendable optics for nearly diffraction-limited focusing of soft x-rays. Opt. Eng..

[24] Niibe M., Sugisaki K., Okada M., Kato S., Ouchi C., Hasegawa T. (2007). Wavefront metrology for EUV projection optics by soft X-ray interferometry in the NewSUBARU. AIP Conf. Proc..

[25] Liu Y., Seaberg M., Zhu D., Krzywinski J., Seiboth F., Hardin C., Cocco D., Aquila A., Nagler B., Lee H.J. (2018). High-accuracy wavefront sensing for x-ray free electron lasers. Optica.

[26] Inoue T., Matsuyama S., Kawai S., Yumoto H., Inubushi Y., Osaka T., Inoue I., Koyama T., Tono K., Ohashi H. (2018). Systematic-error-free wavefront measurement using an X-ray single-grating interferometer. Rev. Sci. Instrum..

[27] Liu Y., Seaberg M., Feng Y., Li K., Ding Y., Marcus G., Fritz D., Shi X., Grizolli W., Assoufid L. (2020). X-ray free-electron laser wavefront sensing using the fractional Talbot effect. J. Synchrotron Rad..

[28] Yamada J., Matsuyama S., Inoue T., Nakamura N., Osaka T., Inoue I., Inubushi Y., Tono K., Yumoto H., Koyama T. (2019). Development of XFEL sub-10 nm focusing mirrors at SACLA: Wavefront-corrected multilayer KB system and upgrade to advanced KB system. Proceedings of the RIAO-OPTILAS-MOPM 2019.

[29] Handa S., Mimura H., Yumoto H., Kimura T., Matsuyama S., Sano Y., Yamauchi K. (2008). Highly accurate differential deposition for X-ray reflective optics. Surf. Interface Anal..

[30] Yashiro W., Takeda Y., Takeuchi A., Suzuki Y., Momose A. (2009). Hard-X-ray phase-difference microscopy using a Fresnel zone plate and a transmission grating. Phys. Rev. Lett..

[31] Takeda M., Ina H., Kobayashi S. (1982). Fourier-transform method of fringe-pattern analysis for computer-based topography and interferometry. J. Opt. Soc. Am..

[32] Bruning J.H., Herriott D.R., Gallagher J.E., Rosenfeld D.P., White A.D., Brangaccio D.J. (1974). Digital wavefront measuring interferometer for testing optical surfaces and lenses. Appl. Opt..

[33] Setson K.A., Brohinsky R. (1985). Electrooptic holography and its application to hologram interferometry. Appl. Opt..

[34] Momose A., Yashiro W., Takeda Y., Suzuki Y., Hattori T. (2006). Phase tomography by X-ray talbot interferometry for biological imaging. Jpn. J. Appl. Phys..

[35] Takeda Y., Yashiro W., Suzuki Y., Aoki S., Hattori T., Momose A. (2007). X-ray phase imaging with single phase grating. Jpn. J. Appl. Phys..

[36] Yashiro W., Takeda Y., Momose A. (2008). Efficiency of capturing a phase image using cone-beam x-ray Talbot interferometry. J. Opt. Soc. Am. A..

[37] Huang L., Idir M., Zuo C., Kaznatcheev K., Zhou L., Asundi A. (2015). Comparison of two-dimensional integration methods for shape reconstruction from gradient data. Opt. Laser. Eng..

[38] Ishikawa T., Tamasaku K., Yabashi M., Goto S., Tanaka Y., Yamazaki H., Takeshita K.T., Kimura H., Ohashi H., Matsushita T. (2001). 1-km beamline at SPring-8. Proc. SPIE.

[39] Bon P., Monneret S., Wattellier B. (2012). Noniterative boundary-artifact-free wavefront reconstruction from its derivatives. Appl. Opt..

[40] Yamada J., Matsuyama S., Sano Y., Yamauchi K. (2017). Simulation of concave–convex imaging mirror system for development of a compact and achromatic full-field x-ray microscope. Appl. Opt..

[41] Yamada J., Matsuyama S., Sano Y., Kohmura Y., Yabashi M., Ishikawa T., Yamauchi K. (2019). Compact reflective imaging optics in hard X-ray region based on concave and convex mirrors. Opt. Express.

[42] Born M., Wolf E. (2001). Principles of Optics.

